# In Situ Cytokine Expression and Morphometric Evaluation of Total Collagen and Collagens Type I and Type III in Keloid Scars

**DOI:** 10.1155/2017/6573802

**Published:** 2017-05-30

**Authors:** Isabela Rios da Silva, Luciana Colombo Rodrigues da Cunha Tiveron, Marcos Vinicius da Silva, Alberto Borges Peixoto, Carla Aparecida Xavier Carneiro, M. A. dos Reis, Pedro Carvalho Furtado, Bárbara Rocha Rodrigues, Virmondes Rodrigues, Denise Bertulucci Rocha Rodrigues

**Affiliations:** ^1^Federal University of Triângulo Mineiro (UFTM) ICBN and CEFORES, Uberaba, MG, Brazil; ^2^Laboratory of Biopathology and Molecular Biology, University of Uberaba (UNIUBE), Uberaba, MG, Brazil

## Abstract

Keloids are characterized by excessive collagen deposition and growth beyond the edges of the initial injury, and cytokines may be related to their formation. The objective of this study was to evaluate the collagen fibers, analyze in situ expression of cytokines in keloid lesions, and compare to the control group. Results showed that there was a predominance of women and nonwhite and direct black ancestry. Keloid showed a significant increase in total and type III collagen. Significantly, the expression of mRNA for TGF-*β* in keloid was increased, the expressions of IFN-*γ*, IFN-*γ*R1, and IL-10 were lower, and IFN-*γ*R1 and TNF-*α* had no statistical difference. Correlations between collagen type III and TGF-*β* mRNA expression were positive and significant, IFN-*γ*, IFN-*γ*R1, and IL-10 were negative and significant, and TNF-*α* showed no statistical difference. We conclude that there was a significant increase of total collagen in keloid and predominance of collagen type III compared to the controls, showing keloid as an immature lesion. There is a significant increase in TGF-*β* mRNA in keloid lesions, and a significant decrease in IFN-*γ* and IL-10, suggesting that these cytokines are related to keloid lesions.

## 1. Introduction

The healing process is performed by a cascade of complex, dynamic, and overlapping events, followed by an inflammatory, proliferative, and remodeling reaction [1]. Changes in these normal processes result in the formation of an exaggerated scar called keloid, characterized by growth of the lesion beyond the initial edges [2] and the nonspontaneous regression over the years [3].

The pathophysiology of keloid is still not fully elucidated yet, although changes in the expression of cytokines [4, 5], increase in fibroblast proliferation [6], and exacerbated collagen synthesis [7] have been described in the literature.

The most frequent types of collagen present in the dermis are collagen type I with 80% and type III with 20% [8]. In the skin, collagen synthesis is performed by fibroblasts, and the genes responsible for the production of collagen type I are COL1A1, located on chromosome 17, which encodes the *α*1(I) chain and COL1A2, located on chromosome 7, which encodes the *α*2(I) chain [9]. Collagen type III is produced by the COL3A1 gene, located on chromosome 2 [10]. Several signaling pathways, such as MAP kinase and NF-kB, induce collagen synthesis by transcription of mRNA, being translated in the RER, hydroxylated, and glycosylated into procollagen [11, 12]. Later, there is excretion into the extracellular medium by exocytosis, where proteolytic enzymes cleave their C and N terminal propeptides, turning it into a tropocollagen. Then, tropocollagens bind together to form collagen fibrils, which give rise to collagen fibers [13]. Type I collagen is considered the mature collagen [14] for being a heterotrimer composed of two identical chains *α*1(I) and one *α*2(I) [15], responsible for the strength and tension of tissues [14]. Type III collagen is an immature collagen; it is a homotrimer, consisting of three *α*1(III) chains [16], synthesized during the early stages of healing [14].

Collagen synthesis can be induced by TGF-*β*, which binds to serine tyrosine kinase ubiquitous receptor (T*β*RII), and then the receptor TGF-*β* I (T*β*RI) is recruited and phosphorylated by T*β*RII. The signal propagates through Smads, a family of intracellular proteins, which, in turn, transport information to the nucleus, stimulating transcription of genes (COL1A1, COL1A2, and COL3A1) and inducing the production of collagens type I and type III [17]. Overexpression of T*β*RI and T*β*RII and increased phosphorylation of Smad proteins were found in keloid fibroblasts, which appear to induce excessive production of collagen [18]. Studies indicate that a failure in eliminating the overexpression of these receptors during the remodeling phase can lead to persistent autocrine effect of TGF-*β* on keloid fibroblasts, causing increased collagen synthesis [19].

However, to maintain a normal healing process, proinflammatory cytokines are required to maintain a balance in this healing process. TNF-*α*, which is important in the early process of skin healing [20], appears to be associated with the suppression of collagen synthesis by fibroblasts [21] and the induction of enzymes that degrade collagen [22]. Similarly, IFN-*γ* appears to inhibit both the proliferation of fibroblasts [23] and the synthesis of extracellular matrix components (MEC) [24]. It has been shown that IFN-*γ* induces activation of Jak/STAT1 [25] pathways and acts at the transcriptional level by inhibiting collagen mRNA synthesis and consequently the formation of collagen fibers [26].

An increase in IFN-*γ*R1 expression seems to be influenced by the interaction between fibroblasts and keratinocytes [27]. The absence of IFN-*γ* receptors has been found in patients with *Schistosoma mansoni* infection and is associated with formation of fibrosis [28]. Polymorphism in the gene of this receptor may be associated with severe liver fibrosis, and it is believed that this receptor may be relevant to the control of fibrosis formation in other diseases [29].

Likewise, IL-10 also appears to have an important role in modulating the healing process, since in vitro studies have demonstrated its role in the induction of collagen synthesis and action of enzymes that degrade collagen, such as MMP1 and MMP8 [30]. Furthermore, IL-10 acts on phosphorylation, via STAT3/AKT signaling, thus inhibiting the collagen synthesis [31]. Thus, this cytokine has been investigated for the treatment of keloids, because intralesional injections with IL-10 in patients with keloids have been performed and reduced the inflammatory process, with a decrease in symptoms and consequently improvement in scar appearance without causing significant side effects [32].

In this context, in the present study, we evaluated the collagen fibers and the in situ expression signature of proinflammatory and anti-inflammatory cytokines in keloid lesions compared to the control biopsies obtained from normal scar samples.

## 2. Material and Methods

### 2.1. Casuistry

We analyzed 73 biopsies, 33 from patients with keloid and 40 from normal scars. The biopsies were performed by the team of plastic surgeons at the Outpatient Clinic Maria da Glória of UFTM. The study included patients diagnosed with keloid, who had abnormal scarring, with growth of the lesion edges beyond the margins of the original scar, and signs and symptoms such as pain, itching, redness, and induration. Patients received corticosteroid (triamcinolone at 20 mg/mL) once a month, and after improvement, patients underwent reconstructive surgery and a fragment of the tissue to be discarded was collected for this study. After surgery, the patients continued with corticoid applications to prevent further recurrences.

Fragments of the normal scar taken from secundiparous or multiparous patients during cesarean section were used as the controls.

We excluded patients who had hypertrophic scars and second intention scars, patients using systemic immunosuppressants, patients with autoimmune diseases, patients with immunodeficiencies of any etiology, or patients who are malnourished. This project was approved by the Research Ethics Committee (CEP) of the Federal University of Triângulo Mineiro (UFTM) under protocol number 45647315.4.0000.5154.

### 2.2. Collection of Material

The material was collected at the outpatient clinic for keloid treatment, in rooms for small surgeries, by the doctors responsible for the sector. The lesion was marked with a surgical pen, and after anesthesia with 2% lidocaine, a cut was made in the lesion with a scalpel, excising the skin with keloid and posterior suture. A part of the fragment was stored in RNAlater (Ambion®) for RT-PCR, and the other part was fixed in buffered formalin for histopathological analysis.

### 2.3. Preparation of Material for Histochemical Analysis

The fragments fixed in 10% formaldehyde were dehydrated in increasing concentrations of alcohol (70 to 100%), diaphanized in xylene and embedded in paraffin. Slides were prepared in 4 *μ*m thick serial sections. Serial sections were performed so as to the slide number 1 was stained with hematoxylin and eosin, the slide number 2 was stained with picrosirius (PS), and the other slides were stored for further analysis. After staining, the slide was mounted with a cover slip and Entellan.

### 2.4. Morphometric Analysis

To quantify the percentage of collagen fibers, the slides stained with PS were analyzed under polarized light at 40x objective (1600x final magnification). To quantify the percentage of collagen fibers, the slides stained by PS were divided into four quadrants and the representative number of measures calculated through the accumulated mean [33]. From this calculation, 10 images per quadrant were analyzed, totaling 40 images per slide, one slide for each case.

The digitized image showed the area consisting of collagen, with birefringence appearance. The analysis of collagen type I was performed by visualization of the red-yellow birefringence; collagen type III, by observing the green birefringence; and total collagen, by marking the two birefringence colors, following protocols established in the literature [34, 35].

For quantification of total collagen and capture of images for differentiation of collagens type I and type III, we used a video camera coupled to a common light microscope with Leica QWin Plus® system (Leica Microsystems Inc., Wetzlar, Germany) installed on a personal computer. The analysis of the percentage of collagen I and III was made through an interactive image analysis system ImageJ® (NIH, Bethesda, Maryland, US).

The maturation index was calculated from the ratio between percentages of collagens type I and type III; values above 1 (one) show the predominance of collagen I, that is, mature, and values below 1 indicate the predominance of collagen type III, with the collagen considered as immature [36].

### 2.5. RNA Extraction

Fragments of the skin with keloid and normal scars stored in RNAlater were ground with a tissue tear and then RNA was extracted using a RNA extraction kit (RNA SV Total RNA Isolation System, Promega, USA) according to the manufacturer's recommendations. After these procedures, the obtained RNA was eluted in 30 *μ*L deionized and RNase-free water for quantification and preparation of complementary DNA (cDNA).

The cDNA was prepared from 1 *μ*g RNA, 0.5 *μ*g Oligo dT (Promega, USA) and autoclaved ultrapure water (Milli-Q). This material was taken to the thermal cycler for a cycle of 5 minutes at 70°C. After immediate cooling, the material was added to dNTP (2.5 mM), M-MLV RT reverse transcriptase (ImProm-II, Promega, USA), and M-MLV-5X reaction buffer (Promega, USA). This reaction was taken to the thermocycler for another 1-hour cycle at 42°C followed by 3 minutes at 10°C. At the end, the material was added to the prepared cDNA 75 *μ*L autoclaved ultrapure water, and samples were then frozen at −20°C until analysis.

### 2.6. Quantitative PCR Reactions (qPCR)

The quantitative mRNA expression of the genes IL-10, TNF-*α*, IFN-*γ*, TGF-*β*, and IFN-*γ*R1 were analyzed by PCR reactions in real time, in cDNA samples of fragment of the skin with keloid and the control scar. The TaqMan system was used in the equipment of PCR in real time (Applied Biosystems, USA) with appropriate primers for such reactions and using *β*-actin as the control. The cDNA synthesized from messenger RNA was used according to the manufacturer's instructions. The results were analyzed based on the value of CT (cycle threshold), and the arithmetic formula to achieve the relative quantification was ∆∆Ct = ∆Ct (treated) − Ct (control) [37].

### 2.7. Immunohistochemistry

Indirect immunohistochemistry was performed in order to evaluate the expression of collagens type I and type III. Deparaffinized sections were treated with 3% hydrogen peroxide in methanol for 10 min for endogenous peroxidase inhibition, incubated for 30 min at 90°C for antigen retrieval, and then incubated with PBS 2% BSA to reduce nonspecific binding. Next, the sections were incubated with monoclonal antibody specific for human anticollagen I (1 : 50; NOVUS, USA; cod-NB600-450) and anticollagen III (1 : 50; Abcam, UK; cod-ab7778). In the second step, a biotinylated Link System (LSAB-K0690, Dako, Carpinteria, CA, USA) was used according to manufacturer instructions. The reaction was visualized by incubating the sections with diaminobenzidine (Sigma, USA) and counterstaining with hematoxylin.

### 2.8. Statistical Analysis

Statistical analysis was performed using the software StatView (Abacus, USA). Assumption of normality of quantitative variables was checked by the Kolmogorov-Smirnov. The variables showed nonhomogeneous distribution or variance and were expressed as median with minimum and maximum values and percentiles and analyzed by the nonparametric Mann–Whitney test. The correlation between two continuous variables with nonnormal distribution was analyzed by the Spearman test (rS). To compare two continuous variables in the same patients, the Wilcoxon test (*U*) was applied. Results were considered statistically significant when the probability was less than 5% (*p* < 0.05).

## 3. Results

We analyzed 73 biopsies, 33 from patients with keloid and 40 normal scars. The average age of patients with keloid was 29.15 ± 16.45 and that of patients in the control group 29.08 ± 7.47 (Table 1).

There was a predominance of women (60.60%), nonwhite (60.60), and patients with direct African ancestry (66.66%). The most common site of the keloid was the earlobe due to piercing/earring perforation (78.78%) (Table 1).

**Figure 1:**
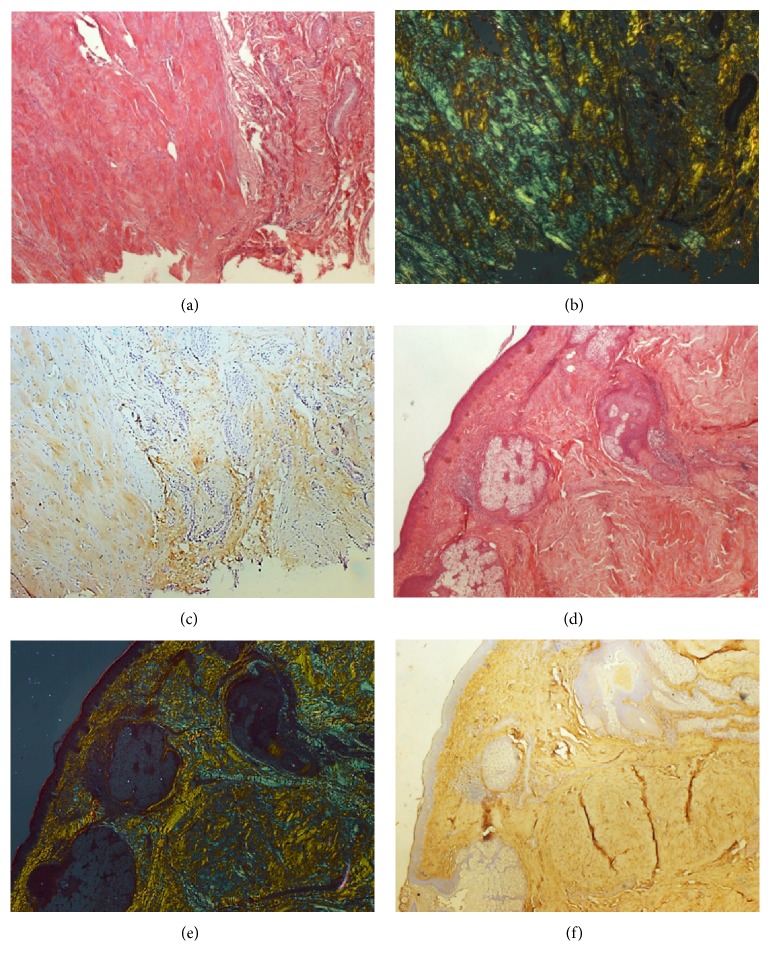
(a) Histological section of keloid stained with picrosirius seen in ordinary light (20x). (b) Histological section of keloid stained with picrosirius seen in polarized light (20x). (c) Immunohistochemistry for type I collagen in keloid (20x). (d) Histological section of keloid stained with picrosirius seen in ordinary light (20x). (e) Histological section of keloid stained with picrosirius seen in polarized light (20x). (f) Immunohistochemistry for type III collagen in keloid (20x).

**Figure 2:**
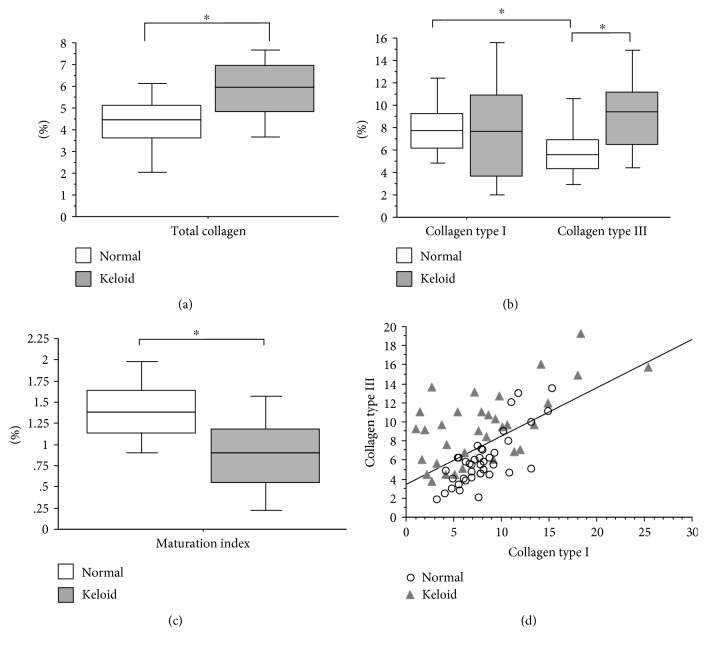
(a) Total collagen percentage present in biopsies of patients with keloid compared with that of patients in the control group (Mann–Whitney; *p* < 0.0001). (b) Percentage of collagens types I and III present in biopsies of patients with keloid compared with that of patients in the control group. Analysis of collagen type I in patients with keloid compared with that in patients in the control group (Mann–Whitney; *p* = 0.653). Analysis of collagen type III in patients with keloid compared with that in patients in the control group (Mann–Whitney; *p* = 0.0001). Analysis of collagen types I and III in the control group (Wilcoxon; *p* < 0.0001). Analysis of collagen types I and III in patients with keloid (Wilcoxon; *p* = 0.126). (c) Maturation index calculated from the percentages of collagen I by III present in biopsies of patients with keloid compared with that of the control group (Mann–Whitney; *p* < 0.0001). The horizontal line represents the median, the bar percentile of 25% to 75% and the vertical line percentile of 10 to 90. (d) Correlation between the percentage of collagens type I and type III in patients with keloid compared with patients in the control group (Spearman; *p* < 0.0001, *z* = 4.293). ∗ indicates significant *p* value.

In both groups, the types of collagen were evaluated by picrosirius staining analyzed under ordinary light, polarized light, and immunohistochemistry (Figures 1(a), 1(b), 1(c), 1(d), 1(e), and 1(f)). There was a significantly higher percentage of total collagen in patients with keloid compared to those in the control group (Figure 2(a)). The analysis of the types of collagen showed that collagen type I of patients with keloids showed no significant difference compared to that of patients in the control group (Figure 2(b)). In turn, collagen type III was significantly higher in patients with keloid compared to those in the control group (Figure 2(b)). The maturation index indicated that biopsies of patients with keloid showed collagen significantly more immature than the control group (Figure 2(c)). The comparison between the types of collagens, in both groups, showed that the percentage of collagen type I was significantly higher compared to that of collagen type III in the control group (Figure 2(b)). And in patients with keloid, the percentages of collagens I and III were similar, with no statistical difference (Figure 2(b)). There was also a significant positive correlation between the percentages of collagens type I and type III in the groups studied (Figure 2(d)).

**Figure 3:**
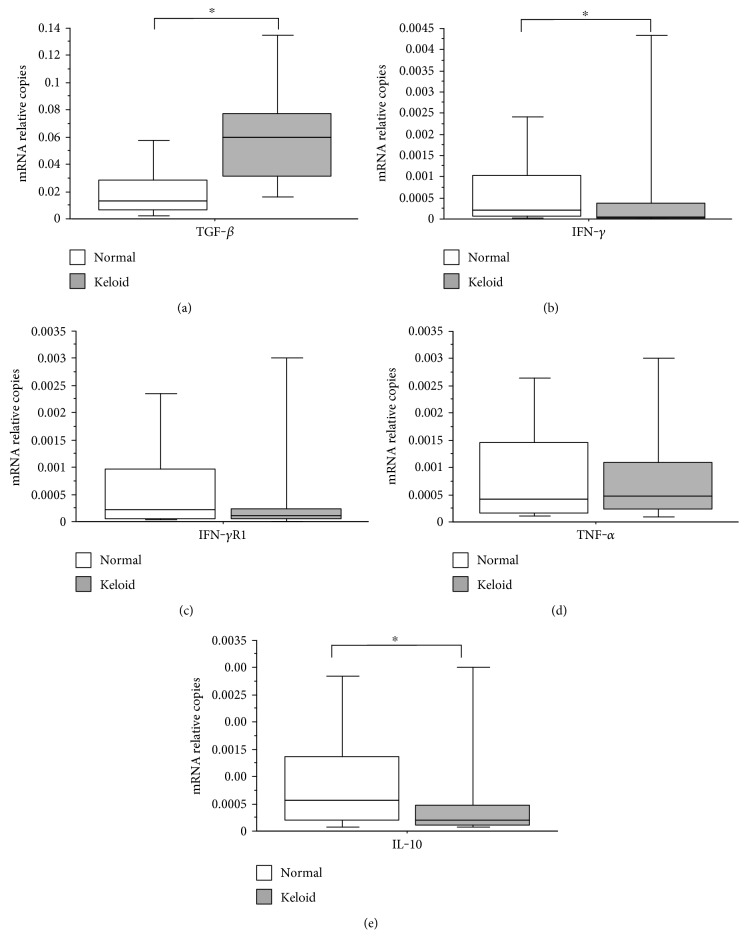
(a) Number of mRNA relative copies for TGF-*β* present in biopsies of patients with keloid compared with that of patients in the control group (Mann–Whitney; *p* < 0.001). (b) Number of mRNA relative copies for IFN-*γ* present in biopsies of patients with keloid compared with that of patients in the control group (Mann–Whitney; *p* = 0.009). (c) Number of mRNA relative copies for IFN-*γ*R1 present in biopsies of patients with keloid compared with that of patients in the control group (Mann–Whitney; *p* = 0.246). (d) Number of mRNA relative copies for TNF-*α* present in biopsies of patients with keloid compared with that of patients in the control group (Mann–Whitney; *p* = 0.911). (e) Number of mRNA relative copies for IL-10 present in patients with keloid biopsies compared to that in patients in the control group (Mann–Whitney; *p* = 0.037). The horizontal line represents the median, the bar percentile of 25% to 75%, and the vertical line the percentile of 10 to 90%. ∗ indicates significant *p* value.

Gene expression analysis of the lesions studied showed that the mRNA expression of TGF-*β* was significantly higher in patients with keloid compared to that in the control group (Figure 3(a)). The expressions of mRNA for IFN-*γ* and IL-10 were significantly lower in patients with keloid compared to the controls (Figures 3(b) and 3(e)).

Regarding the analysis on the relative number of mRNA copies of IFN-*γ*R1, patients with keloid had a lower relative number of copies of mRNA when compared to the controls, but without significant differences (Figure 3(c)). When analyzing the relative number of copies of mRNA for TNF-*α*, it shows no significant difference between patients with keloid compared with those in the control group (Figure 3(d)).

When comparing the percentage of type I collagen and the number of mRNA copies of TGF-*β*, IFN-*γ*, IFN-*γ*R1, TNF-*α*, and IL-10 between the groups, there was no significant correlation (data not shown).

**Figure 4:**
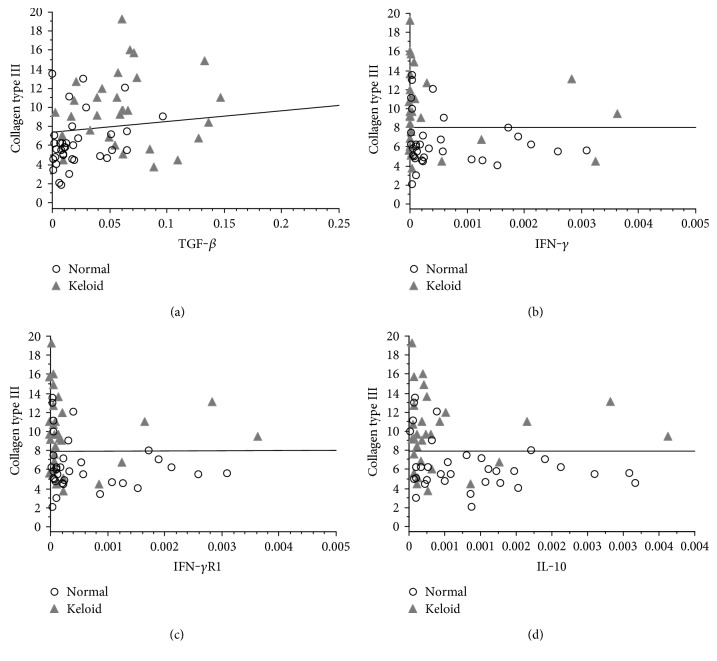
(a) Correlation between the percentage of type III collagen and the number of mRNA relative copies for TGF-*β* in patients with keloid compared with that in the control group (Spearman; *p* = 0.001, *z* = 3.210). (b) Correlation between the percentage of type III collagen and the number of mRNA relative copies for IFN-*γ* in patients with keloid compared with that in patients in the control group (Spearman; *p* = 0.015, *z* = −2.425). (c) Correlation between the percentage of type III collagen and the number of mRNA relative copies for IFN-*γ*R1 in patients with keloid compared with that in patients in the control group (Spearman; *p* = 0.021, *z* = −2.303). (d) Correlation between the percentage of type III collagen and the number of mRNA relative copies for IL-10 in patients with keloid compared with that in patients in the control group (Spearman; *p* = 0.014, *z* = −2.445).

However, when analyzing the ratio between the percentage of collagen type III and TGF-*β* mRNA expression between the groups, there was a significant positive correlation (Figure 4(a)). In contrast, a significant negative correlation was found between the percentage of collagen type III and mRNA expression of IFN-*γ*, IFN-*γ*R1, and IL-10 between the groups (Figures 4(b), 4(c), and 4(d)).

On the other hand, there was no significant correlation when comparing the percentage of collagen type III and the number of mRNA copies for TNF-*α* between the groups (data not shown).

## 4. Discussion

In the present study, we evaluated the percentages of total collagen and type I and type III collagens and the relative number of mRNA copies for TGF-*β*, IFN-*γ*, IFN-*γ*R1, TNF-*α*, and IL-10 in keloid fragments compared to normal scars.

The average age of patients with keloid, in this study, was 29.15 ± 16.45 years, predominantly women (60.60%) and nonwhite (60.60%). Similar to our results, the literature shows that patients with keloid present an average age between 24 and 35.7 years [3, 38] and the appearance of these lesions occurs between 11 and 40 years [39]. It has been suggested that this age group is associated with hormonal changes, surgery, trauma [40], increased exposure to lesions by perforations, such as earrings/piercings [41], and also a higher recurrence during pregnancy [42]. In our study, there was a prevalence of nonwhite, which has also been reported in the literature [41, 43], and studies have been conducted to prove the presence of genetic factors from family heredity and the frequency in specific ethnic populations [43, 44].

Herein, total collagen was significantly higher in patients with keloid compared to those in the control group. Studies show that the keloid fibroblasts produce more collagen than normal skin fibroblasts [45] and keloid fragments have a greater volume of total collagen density when compared to the control group [46]. Our results are consistent with the literature [47, 48], in which the increase of collagen may be related to several factors. It is believed that, in keloid lesions, there is a decreased production of metalloproteinases (MMPs) [49] and an increased synthesis of inhibitors of metalloproteinases (TIMPs), thus deregulating the degradation process of excessive collagen [50]. In addition, the excess in collagen synthesis is associated with changes in the expressions of some genes [51] and reduction in apoptotic activity caused by mutation in p53 when compared to normal skin fibroblasts [52, 53]. In this sense, several factors may be involved in the excessive deposition of collagen formed in keloid.

In our study, performed on biopsies from keloid lesions and normal scars, we detected a significant increase in type III collagen (immature collagen) in patients with keloid compared those in the control group. A reduction in the cross-links of type III collagen fibers in keloids appears to interfere with the composition of MEC, hindering maturation and the reestablishment of scar stability, and leading to an increase in the synthesis of collagen type III [54]. In experimental tubulointerstitial nephritis, it has been shown that excess type III collagen is formed by myofibroblast, and this cell appears to be responsible for the increased synthesis of *α*1(III) mRNA, thus contributing to the development of fibrosis [55]. TGF-*β*1 has been pointed as an important growth factor in the differentiation of fibroblasts into myofibroblasts [56], and this cytokine also appears to inhibit apoptosis mechanisms by activation of P13K/AKT signaling pathways, thus keeping these myofibroblasts at profibrotic activity [57]. Studies also demonstrate that fibroblasts isolated from keloid are more sensitive to activation by TGF-*β* than normal skin fibroblasts [58]. Thereby, several factors may be associated with excessive synthesis of type III collagen synthesis in keloids and TGF-*β* may be an important factor in this stimulus, since, in our study, we found a significant positive correlation between this cytokine and type III collagen.

Moreover, in our results, the expression of mRNA for IFN-*γ* was significantly lower in patients with keloid. The antifibrotic action of IFN-*γ* has been investigated in renal [59] and hepatic [60] diseases and also in keloids [61]. In culture of keloid fibroblasts, it has been demonstrated that different doses of IFN-*γ* showed no antagonistic activity to the fibrotic effect of TGF-*β* in the keloid fibroblasts [5]. In experimental models, IFN-*γ* has been found to inhibit both fibroblast proliferation at the site of the lesion [24] and the collagen synthesis [62]. Still, patients with keloid treated with intralesional recombinant IFN-*γ* showed a reduction in lesion size, reducing the number of collagen bundles and fibroblastic activity, and increased inflammatory process [63]. Nevertheless, despite the antifibrotic effects, some patients have reported a mild headache with the application of intralesional IFN-*γ* [64]. IFN-*γ* signaling begins with its binding to the respective receptors, and, in this study, we found a decrease in IFN-*γ*R1 mRNA expression but with no significant difference. The literature still lacks a relationship of IFN-*γ*R1 in keloids. However, in severe hepatic fibrosis associated with *Schistosoma mansoni* infection, polymorphism in the gene encoding IFN-*γ* R1 was demonstrated, and the authors suggest that this receptor is one possible candidate genes in the control of other abnormal fibrotic diseases [29]. Thus, we believe that a simultaneous reduction in mRNA expression for IFN-*γ* and IFN-*γ* R1 can be also contributing to the formation of keloids; in our study, we found a significant negative correlation between the percentage of collagen type III and the number of copies of mRNA for IFN-*γ* and IFN-*γ*R1.

In the present study, we found no significant difference in the relative number of mRNA copies for TNF-*α* in the groups studied. At the initial stages of healing, TNF-*α* plays a key role in the recruitment of inflammatory cells to the site of the lesion [65] and still appears to interfere with type I collagen degradation in normal skin fragments, increasing the collagenolytic activity of MMP-1 [22]. In culture of human fibroblasts, it was demonstrated that TNF-*α* moderately inhibits both collagen gene transcription and synthesis [66]. Thus, we found no studies on TNF-*α* associated with keloids and although this cytokine is important in the healing process, its role in keloid is not well elucidated.

Furthermore, in this study, patients with keloid showed mRNA expression for IL-10 significantly lower compared to the controls. We did not found any previously study associating the expression of IL-10 with keloids; however, in culture of fibroblasts from patients with scleroderma and normal skin, stimulated with IL-10, there was a decrease in collagen *α*1(I) mRNA expression in both groups. The authors believe that IL-10 might influence mRNA expression of MEC components and further modify the development of tissue fibrosis [67]. As it is an anti-inflammatory cytokine that plays an important role in the healing process, lesions induced in IL-10 knockout animals showed increased inflammatory response and excessive deposition of collagen compared to normal animals. In this way, intralesional applications of this cytokine were made in humans, which resulted in an improved scarring and reduced redness of the lesion, indicating that this cytokine may be an alternative therapy to minimize and/or a prophylaxis of exacerbated healing [32]. Thereby, our findings indicate that IL-10 may play a role in the pathogenesis of keloids with negative correlation between the collagen type III and IL-10 expression can be related to this low IL-10 expression in keloids.

The results presented here point to a complex relationship between cytokine balance and collagen synthesis and maturation. Furthermore, it stressed out that intervention in this balance may be useful as a therapeutical tool in the management of keloid scar in susceptible subjects.

## 5. Conclusion

In summary, in this study, patients with keloid showed an increase in total collagen with predominance of collagen type III compared to normal scars, showing that keloid can be considered an immature lesion. Also, keloid lesions were associated with a significant increase of TGF-*β* mRNA and the decrease in IFN-*γ* and IL-10 mRNA, suggesting that these cytokines might be related to the development of keloid lesions.

## Figures and Tables

**Table 1:** General and clinical characteristics of patients with keloid scars.

Total biopsies	33
Mean age	29.15 ± 16.45
Gender (female)	20 (60.60%)
Nonwhite	20 (60.60%)
Black ancestry	22 (66.66%)
Positive family history	13 (39.39%)
Anatomical location of the biopsies	
Earlobe	26 (78.78%)
Abdomen	5 (15.15%)
Chest	2 (6.06%)
Cause of keloid	
Ear piercing	26 (78.78%)
Surgery	6 (18.18%)
Acne	1 (3.03%)
